# Supplementary material for: Genome-resolved analyses show an extensive diversification in key aerobic hydrocarbon-degrading enzymes across bacteria and archaea
Source: BMC Genomics. 2022 Oct 6;23:690. doi: 10.1186/s12864-022-08906-w (PMC9535955; doi:10.1186/s12864-022-08906-w)
Supplement: Supplementary file 9 — Additional file 9: Supplementary Figure S1. Schematic representation of HC degradation pathways studied in this work. Purple circles show key HC degrading enzymes trigerring the degradation. Blue circles are other crucial enzymes. Important intermediate compounds are written in blue. Supplementary Figure S2. Distribution of 143512 genomes of the GTDB database release 89 in different phyla. Supplementary Figure S3. Distribution of aliphatic hydrocarbon-degrading genes across domain bacteria at the phylum level. In plot A, the color gradient indicates the proportion of degrading members of each phylum to the entire HC degrading community. In plot B, the color gradient shows the percentage of HC degrading members of each phylum. Columns are the name of genes involved in HC degradation, which key ones are represented in red. Supplementary Figure S4. Distribution of aromatic hydrocarbon-degrading genes across domain bacteria at the phylum level. In plot A, the color gradient indicates the proportion of degrading members of each phylum to the entire HC degrading community. In plot B, the color gradient shows the percentage of HC degrading members of each phylum. Columns are the name of genes involved in HC degradation, which key ones are represented in red. Enzymes written in blue are shared among the degradation processes of different aromatic compounds (xylene, phenol and naphthalene). Supplementary Figure S5. Distribution of aliphatic hydrocarbon-degrading genes across domain archaea at the phylum level. In plot A, the color gradient indicates the proportion of degrading members of each phylum to the entire HC degrading community. In plot B, the color gradient shows the percentage of HC degrading members of each phylum. Columns are the name of genes involved in HC degradation, which key ones are represented in red. Supplementary Figure S6. Distribution of aromatic hydrocarbon-degrading genes across domain archaea at the phylum level. In plot A, the color gradient indic [file 12864_2022_8906_MOESM9_ESM.docx]

**Supplementary Figures**

**
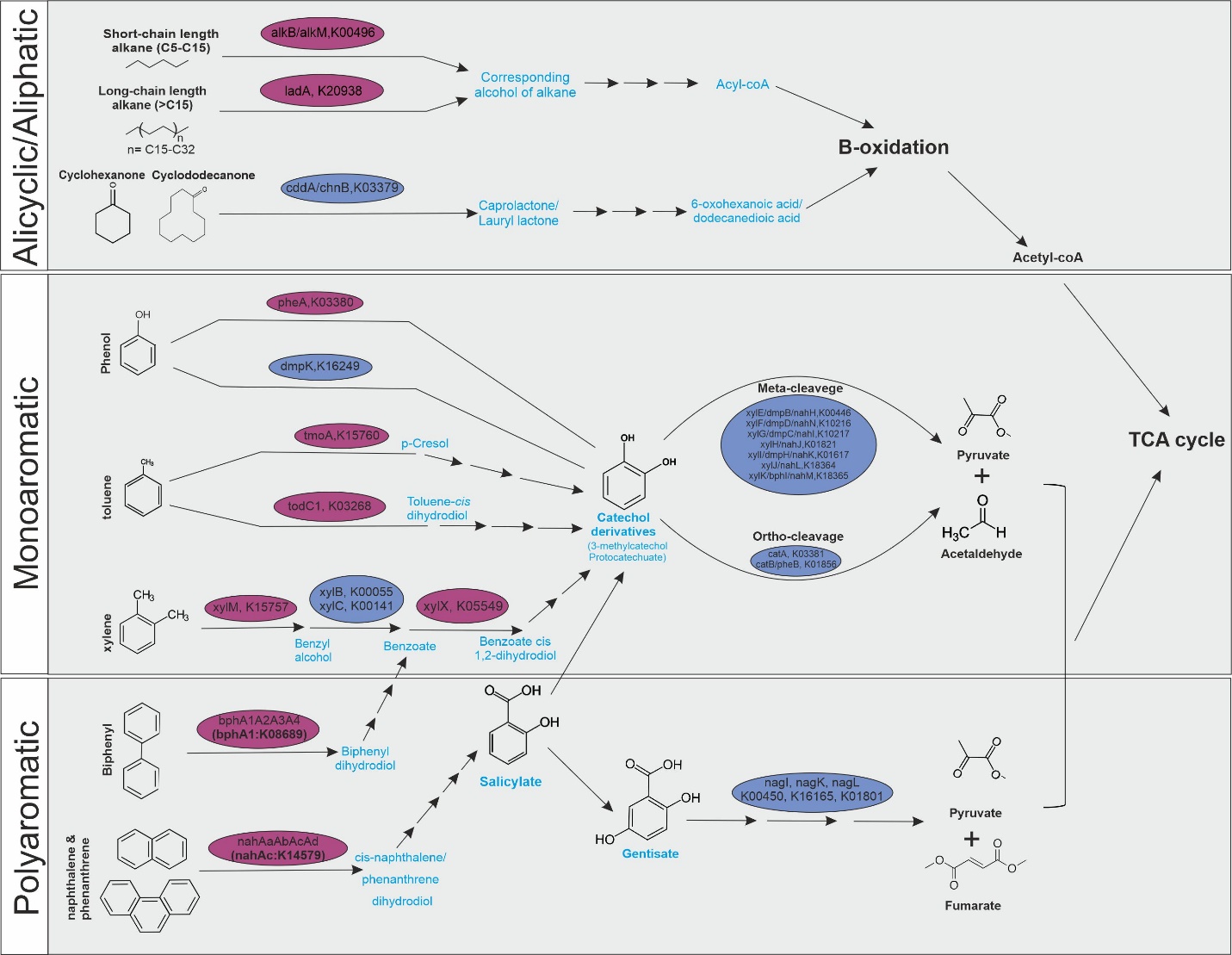
**

**Supplementary Figure S1-** Schematic representation of HC degradation pathways studied in this work. Purple circles show key HC degrading enzymes trigerring the degradation. Blue circles are other crucial enzymes. Important intermediate compounds are written in blue.


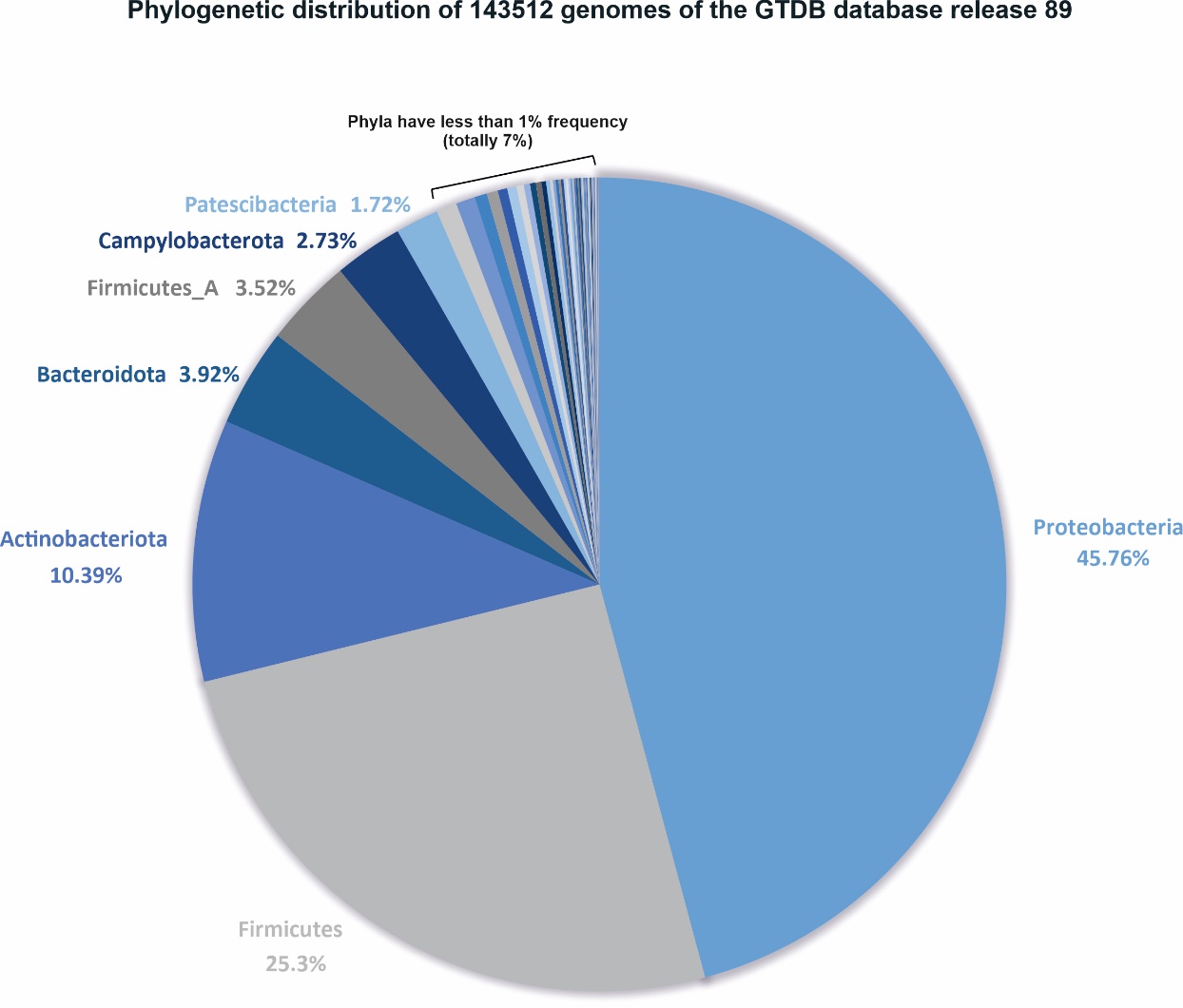


**Supplementary Figure S2-** Distribution of 143512 genomes of the GTDB database release 89 in different phyla.


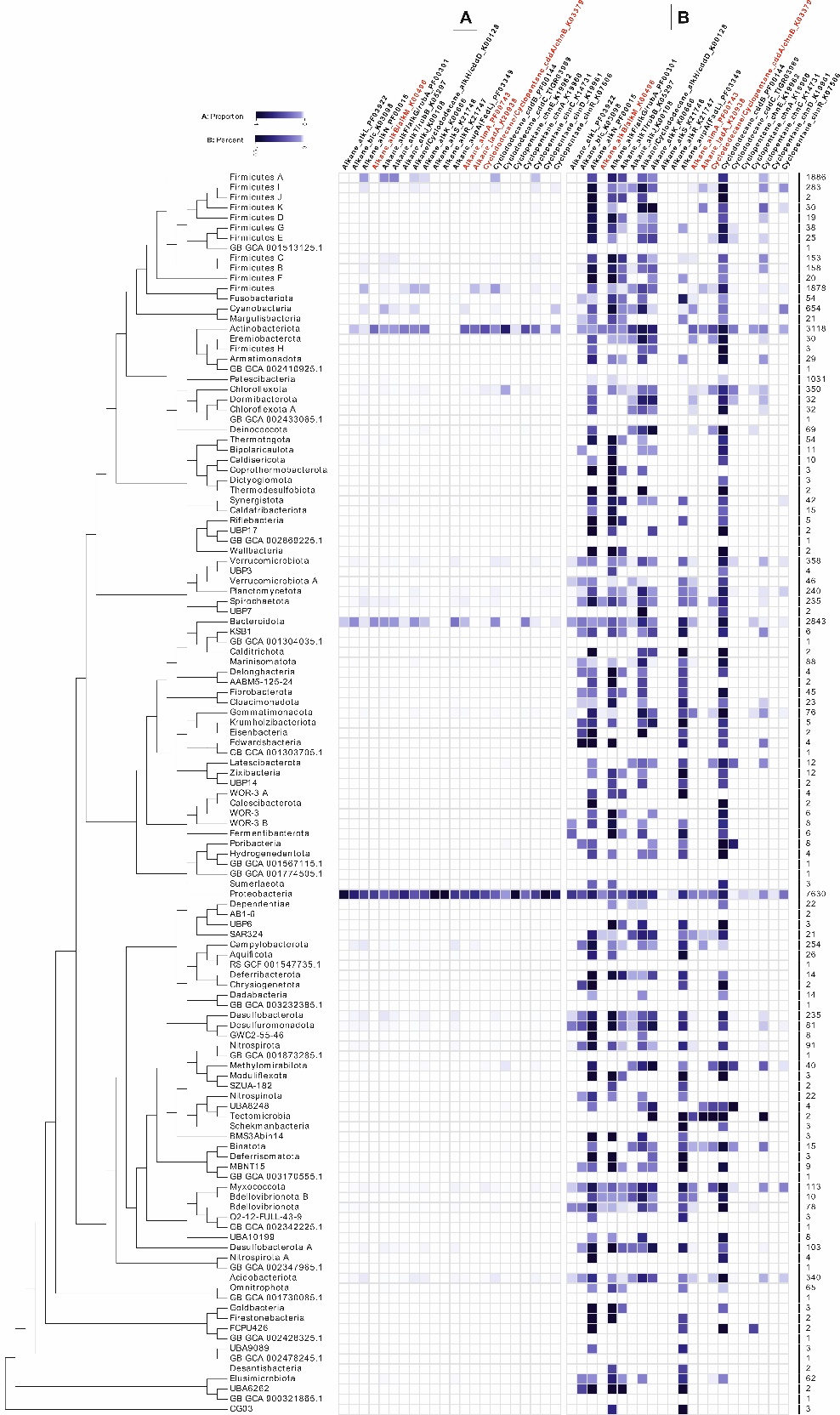


**Supplementary Figure S3-** Distribution of aliphatic hydrocarbon-degrading genes across domain bacteria at the phylum level. In plot A, the color gradient indicates the proportion of degrading members of each phylum to the entire HC degrading community. In plot B, the color gradient shows the percentage of HC degrading members of each phylum. Columns are the name of genes involved in HC degradation, which key ones are represented in red.


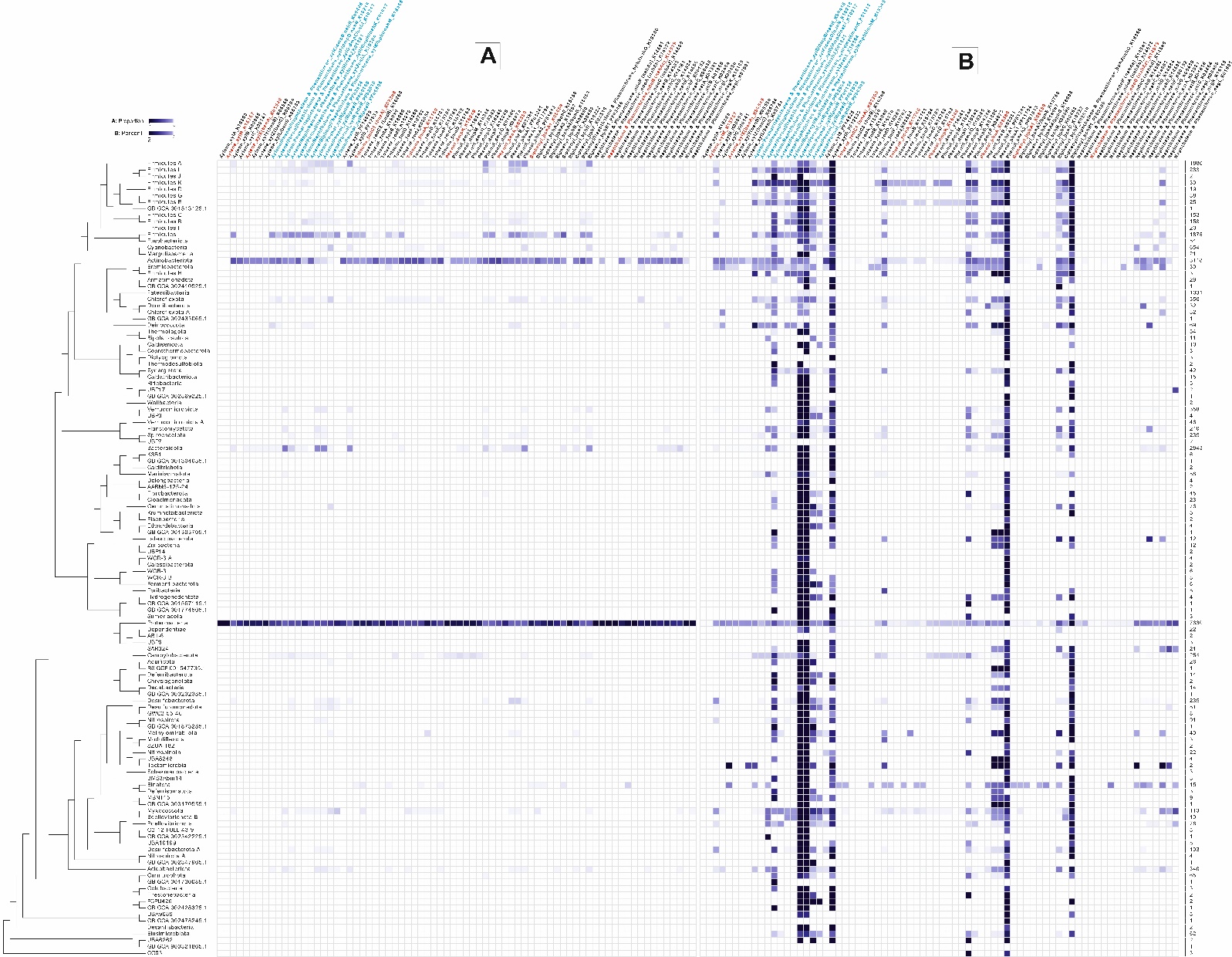


**Supplementary Figure S4-** Distribution of aromatic hydrocarbon-degrading genes across domain bacteria at the phylum level. In plot A, the color gradient indicates the proportion of degrading members of each phylum to the entire HC degrading community. In plot B, the color gradient shows the percentage of HC degrading members of each phylum. Columns are the name of genes involved in HC degradation, which key ones are represented in red. Enzymes written in blue are shared among the degradation processes of different aromatic compounds (xylene, phenol and naphthalene).

**
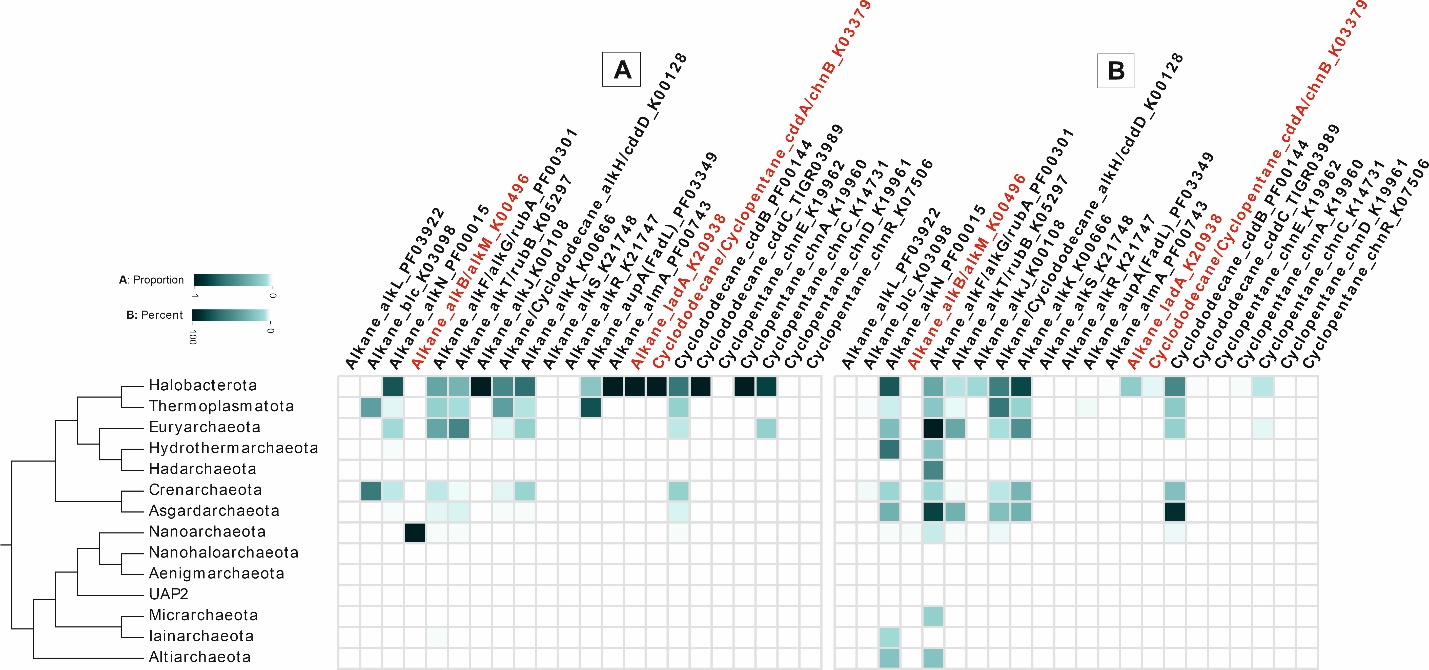
**

**Supplementary Figure S5-** Distribution of aliphatic hydrocarbon-degrading genes across domain archaea at the phylum level. In plot A, the color gradient indicates the proportion of degrading members of each phylum to the entire HC degrading community. In plot B, the color gradient shows the percentage of HC degrading members of each phylum. Columns are the name of genes involved in HC degradation, which key ones are represented in red.


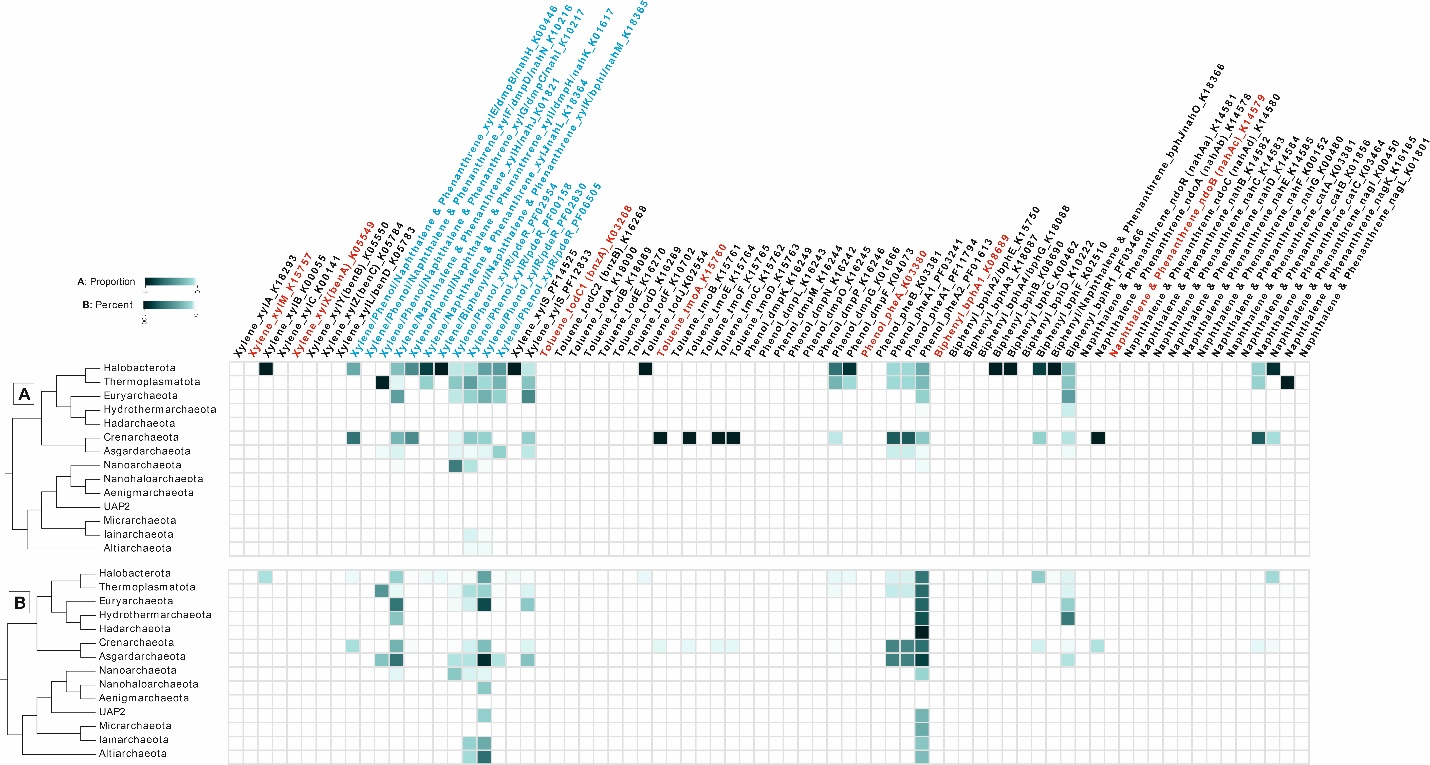


**Supplementary Figure S6-** Distribution of aromatic hydrocarbon-degrading genes across domain archaea at the phylum level. In plot A, the color gradient indicates the proportion of degrading members of each phylum to the entire HC degrading community. In plot B, the color gradient shows the percentage of HC degrading members of each phylum. Columns are the name of genes involved in HC degradation, which key ones are represented in red. Enzymes with blue color are shared among the degradation processes of different aromatic compounds (xylene, phenol and naphthalene).


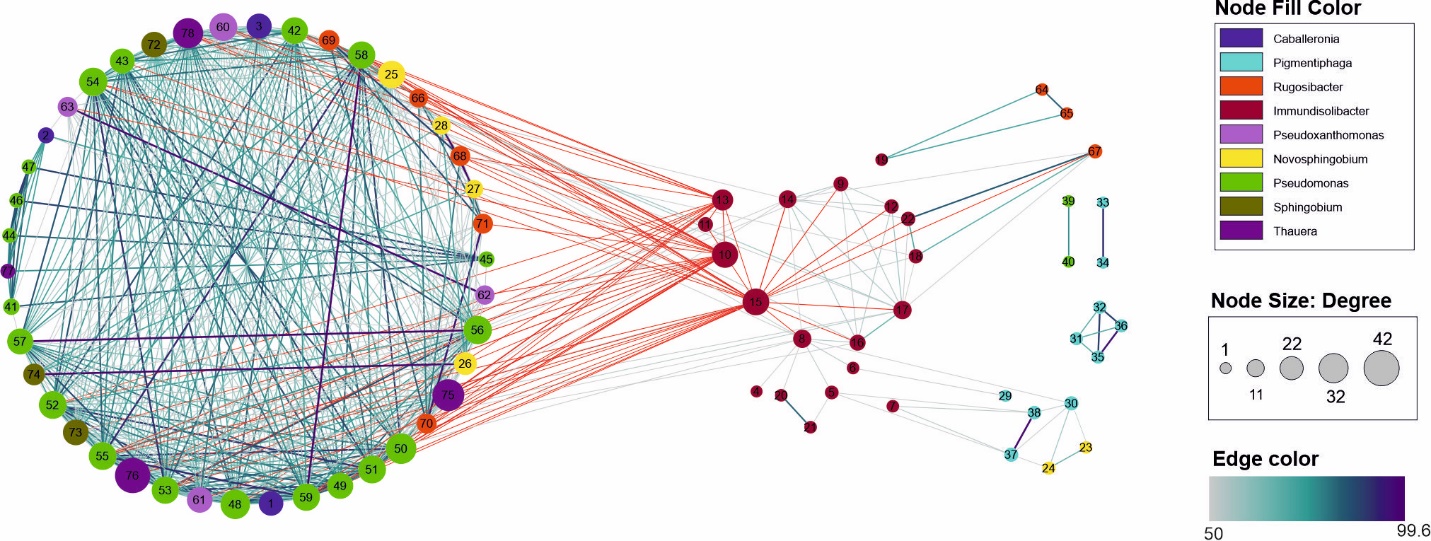


**Supplementary Figure S7-** Network interaction between 18 copies of xylX gene in *Immundisolibacter cernigliae* and other genomes with more than two copies of this gene. Only the blast identity values between 50 to 100 percent are shown. Edges are color-coded based on their blast identity. The size of nodes is based on the “Degree,” which is determined by the number of edges of each node. Edges in red are versions of xylX in *Immundisolibacter cernigliae* that had a higher degree than others. The gene ID of the assigned number of each node is represented in Supplementary Table S7.

**
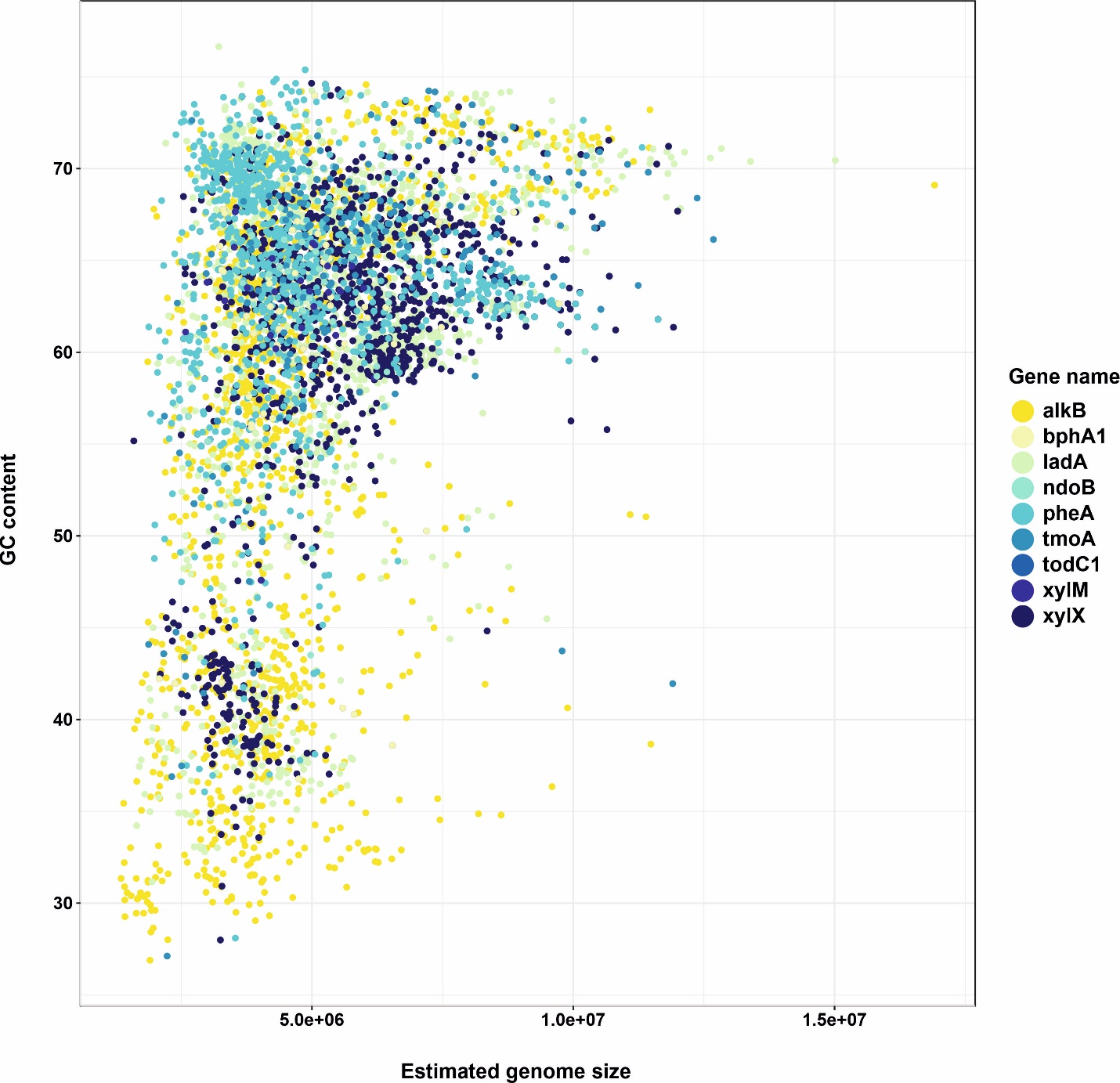
**

**Supplementary Figure S8-** Distribution of genome size versus GC content of the studied genomes with key HC degrading genes.


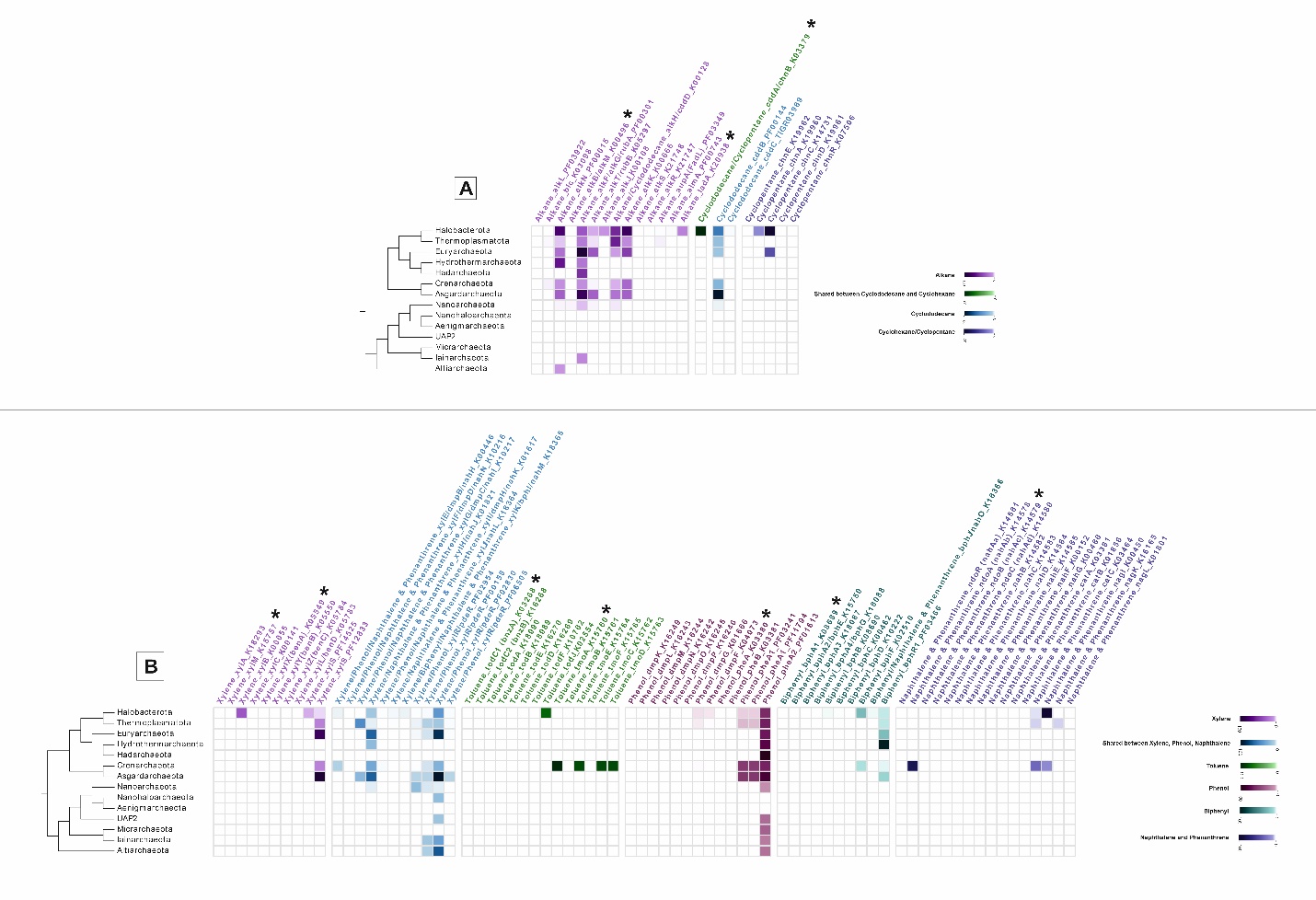


**Supplementary Figure S9-** Distribution of aliphatic (A) and aromatic (B) hydrocarbon-degrading genes across domain archaea at the phylum level. Columns show the name of genes involved in HC degradation and are represented in different colors for various compounds. The color gradient for genes of each compound indicates the percentage of HC degrading members of each phylum.


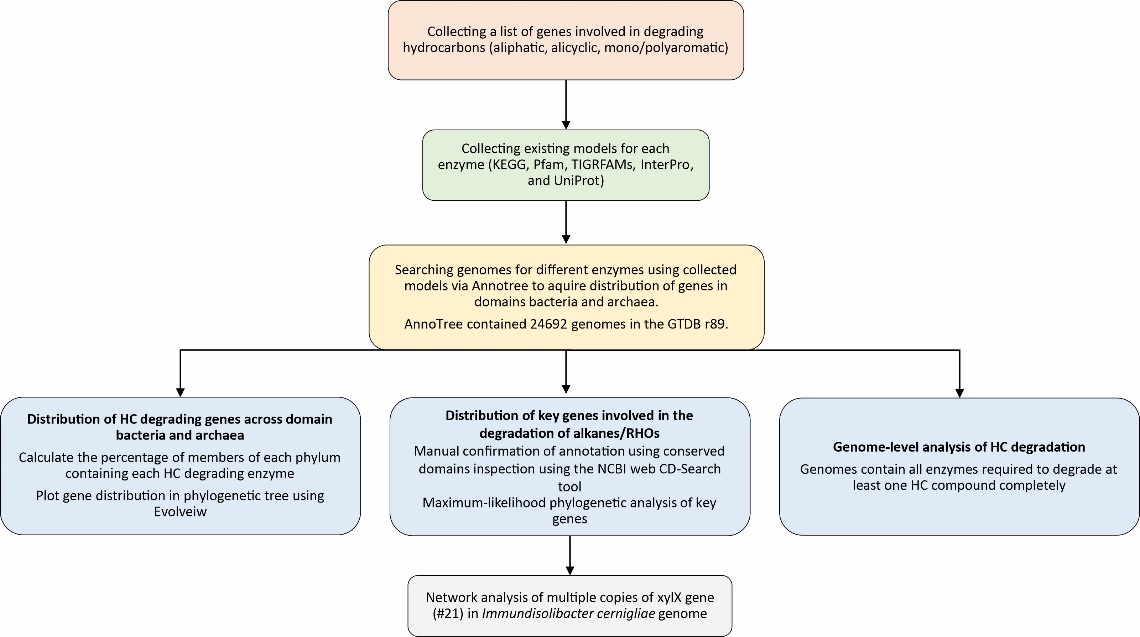


**Supplementary Figure S10-** The overview of workflow that has been done in the present study**.**
